# Iranian school-aged twin registry: preliminary reports and project progress

**DOI:** 10.1186/s12887-023-03865-x

**Published:** 2023-02-10

**Authors:** Hamidreza Abtahi, Marsa Gholamzadeh, Roza Baharii

**Affiliations:** 1 Pulmonary and Critical Care Medicine Department, Thoracic Research Center, Imam Khomeini Hospital Complex, Tehran University of Medical Sciences, Tehran, Iran; 2 Health Information Management and Medical Informatics Department, School of Allied Medical Sciences, Tehran University of Medical Sciences, Tehran, Iran; 3 Thoracic Research Center, Imam Khomeini Hospital Complex, Tehran University of Medical Sciences, Tehran, Iran; 4 Cancer Research Center, Cancer Institute of Iran, Tehran University of Medical Sciences, Tehran, Iran

**Keywords:** School-aged twin, Registry, Zygosity, Database

## Abstract

**Background:**

National Persian school-aged twin registry was established to provide a platform for twin studies. In this report, we describe defining registry characteristics, database design, and preliminary results regarding gathered data in the first phase of the registry program.

**Method:**

Through focus group discussions, the required data elements to design the database and data collection process were defined. First, a list of twins in school-aged groups was retrieved from the electronic database of the Ministry of Education. Tehran schools were selected for the first phase of our registry. Standard “Pea-in-Pods” questionnaire and twins’ similarity questionnaires were filled out by the parents themselves in addition to demographic information. Data were analyzed using SPSS v.22.

**Results:**

The first national school-aged twin registry was established in 2018. Firstly, the required data sets and data collection process were defined using focus group discussions. At the country level, the initial information on 189,738 students was retrieved from the national database of the Ministry of Education. They were born between 2003 and 2017, of which 94,997 are boys (50.1%) and 94,741 are girls (49.9%). Of them, a total of 5,642 pairs of school-aged twins participated in the first phase of our program. Our sample size comprised 9772 twins, 906 triples, and 92 quadruplets. The analysis of the zygosity questionnaire showed that 14% of twin pairs were identified as monozygotic twins.

**Conclusion:**

Recruiting school-aged twins through school health assistants leads to high enrollment and decreasing costs for the twin registry. The study showed a high rate of dizygotic twins that need to be verified by twin bio-sample in the next phase of studies.

**Supplementary Information:**

The online version contains supplementary material available at 10.1186/s12887-023-03865-x.

## Introduction

Twin-based studies are a valuable means to study the effect of genetic and environmental factors on health and disease [1]. Although identical monozygotic (MZ) twins have a similar genetic background, fraternal or dizygotic (DZ) twins share only fifty percent of their genes that usually live in the same environment. So, twin researchers can differentiate the role of genetic and environmental effects in such domains as intelligence, individual and social behavior, nutritional habits, somatic, and mental disorders. The twin registry is usually implemented at the national level which can provide a valuable database for an epidemiological study [2–4]. One of the first founded twin registries in the world is the Danish Twin Registry (DTR) which has known as the basis of many population-based cohort studies [5, 6]. After that national twin registries have been developed all over the world to make an increasing amount of data available [7].

Two of the main challenges of twin registries are the high cost of data entry and twin loss to follow-up [8–10]. The continuity of twins appearing in such research programs may vary from a few months to several years, depending on the amount of research funding and the validity of the ethical code [10, 11].

Although local twin registries have been developed in Iran since the mid-2000s by different researchers [12], the continuity of twin recruitment could not be achieved in these registries due to resource limitations. So, the National Persian twin registry was established in 2016 for all age groups supported by the Iranian Ministry of Health [9]. Due to limited enrollment of adult twins, the Persian twin registry continued in the school-aged group through schools and newborns through hospitals since 2018 [9, 13, 14]. In school-aged twins, the challenges of high registration costs and continuity of twins’ participation could be addressed with the aid of health assistants who work in schools. In this report, we describe defining registry characteristics, database design, and preliminary results regarding gathered data in the first phase of the school-aged twin registry program.

In the school-aged group, we also expected that due to the upward use of assisted reproductive technologies (ART) techniques in Iran since its establishment since 1984 has been associated with a significant increase in multiple dizygotic twins [15, 16].

## Methods

This study is a descriptive qualitative study to describe the main characteristics of the registry and preliminary reports in the first phase. The details of the data collection method, school-aged twins’ enrollment process in the first phase, and the required data elements that should be recorded were determined using focus group discussions with experts. The initial analysis of gathered data is described in the following.

### Target group

School-aged twins were selected as the target group for this plan. The reasons mentioned by researchers for choosing this age group included the following:

1. Prevention and promotion programs are more crucial for children and adolescents due to the psychosomatic challenges of high growth rate and puberty.
2. Most twins at this age live in a similar environment in the same family, which allows examination of genetics and micro environmental factors such as the effect of different friends, food habits, and social interests.
3. Twins' recruitment and follow-up are more easily performed through their schools and their parents in school-aged twins.

The study will exclude participants if they would not available for long-term follow-up. Twin pairs younger than six years or older than 18 years old and those who are unable to provide consent were excluded too. Twins who one of them is not available or one of them was dead will also be excluded.

### Determining our program characteristics and national database datasets

Since a comprehensive program is needed to set up a population-based study, focus group discussions were used to identify the target group, determine the required minimum datasets for the development of the national twin database, specify questionnaires, and data recording process. Following the establishment steering committee, the data items and enrollment process were identified through focus group (FG) discussions with experts. FG discussions are the best solution to gather in-depth information about experts' thoughts and opinions on a topic.

Experts were selected through a purposive sampling technique for focus group discussions. All of the experts who have experience in twin studies were invited to participate in these meetings. To schedule sessions, a Ph.D. candidate, (MG) who has expertise in conducting qualitative studies contacted all of the experts by telephone conversation to invite them. She conducted all sessions and she explained the main objective of the sessions was explained to all participants at the beginning of the first session. Then, they discussed about required data elements that should be gathered through this survey.

Eight sessions were held over six months in the Thoracic Research Center, Imam Khomeini Hospital complex. A suggested list of data elements was formed through 90 min of focus group discussions [17]. In these meetings, important points were taken notes by the conductor. Thematic analysis as a qualitative analysis technique was employed to analyze the focus group discussion outcomes. Qualitative analysis was conducted to determine the main domains and sub-domains of data elements. First, transcripted gathered data was checked by other researchers. Subsequently, initial codes were assigned manually to transcripted data according to their interpretations by one researcher. All the coded data are coded based on the degree of similarity to create the final coding list. The main themes were extracted from the code lists and validated by all experts.

All of the data elements were classified into main themes and sub-themes. Expert consensus was reached when ≥ 85% of experts either agreed or disagreed with data elements. The Consolidated criteria for reporting qualitative research (COREQ) checklist was used to report the results. The result of the focus group discussions including the enrollment process is described in the result section.

### School-aged twins’ enrollment and registration

In collaboration with the Ministry of Education of Iran, the complete list of school-aged twins, their city of residency, and their schools was retrieved from the national electronic educational system. Since the affiliation of researchers to Tehran University of Medical Sciences, we collaborated with Tehran Education Organization to recruit volunteered twin students who lived in Tehran city. At this phase of the project, the Ministry of Education did not provide us with contact information for the twins but provided us with the population of twins and their school names in different regions of Tehran. After holding educational sessions for school health experts, schools contacted twin families to inform them about our program. Data collection is ongoing and currently in its fifth year.

**Fig. 1:**
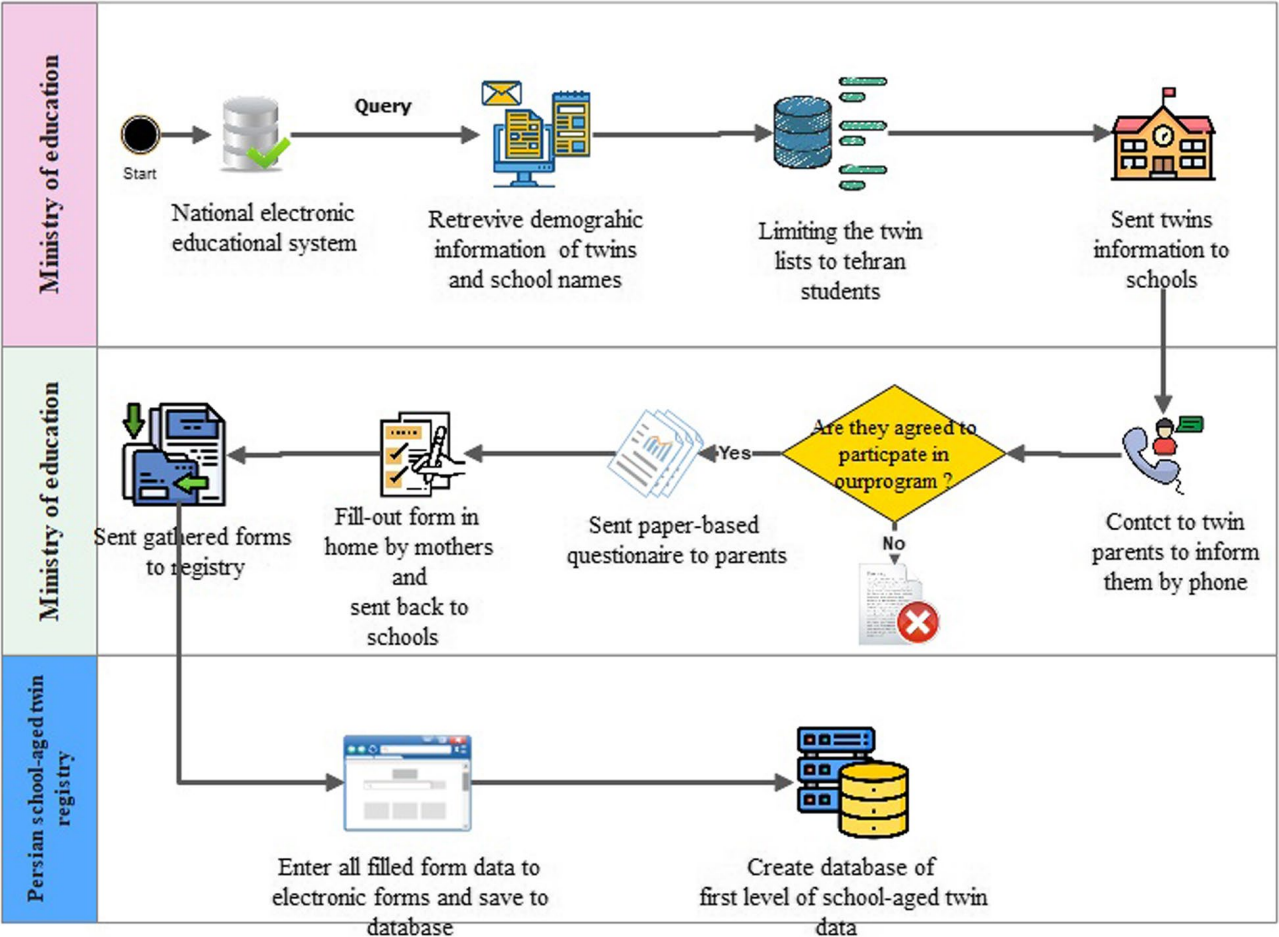
Process of data collection method

Since participation in this project was voluntary, complete information regarding the program was sent for parents to invite them with aid of the twins’ school. As part of the stage of contacting parents, the parents of the twins were offered to express their readiness and the paper-based consent form was passed to parents in-hand by schools to get their permission to enroll their children. After obtaining informed consent, the relevant paper-based questionnaire was filled out by the parents themselves. The process of the data collection method is described in Fig. 1.

A questionnaire included the twins’ and their parent's information, zygosity data, and informed consent for participating in our program filled out by the twins' mothers at home with aid of their twins. The first part of the questionnaire comprises twins' and their parents’ demographic data, urban area, national code, and contact information. The health staff of the Tehran Educational organization was available by phone to support parents with any questions regarding the aim of the study and how to fill out this questionnaire.

### The platform for recording information and informing twins

As the Iranian school-aged twin registry was founded, the school-aged twin registry website (www.persiantwin.ir) was created to develop a platform for organizing and recording the twin’s information. An informational Website was also designed for this project. Some additional sections were assumed to inform the twins' families and how to participate in the twin's health follow-up program. Twins can connect with our registry and gain essential information using the website and/or directly by phone. They can make sure that the validity of our program. It was implemented in Persian to create better communication between our project and the twin family. Also, all the information collected by paper questionnaires was entered on the website through an electronic form.

### Zygosity and the physical similarity questionnaires of twin pairs

Though the best method for zygosity determination can be obtained with DNA markers, it is so expensive and time-consuming in large population-based epidemiological studies. Thus, we used a standard questionnaire-based survey. Our questionnaire consists of two parts.

The first part consists of the three-item questionnaire known as the “Pea-in-Pods” questionnaire for twins’ mothers which has known as the most reliable questionnaire for zygosity determination with 95% accuracy [18]. The scores for each question were defined based on the study conducted by Ooki et al. [19]. The assigned scores for each question are described in the following. According to the first question, if the mother's answer to the first question is that the twins are like two halves of an apple, a score of one is assigned to this question. If the answer is that they are like two ordinary siblings, two points should be assigned to this question. A score of one to three was assigned to the second question based on the degree of difference and confusion between the twins. In the third question, based on who confuses the twins, a score of one to four was assigned [20].

Moreover, the second part of our questionnaire included the 11-items to investigate the physical similarity of twin pairs. The second part of the questionnaire was adapted from the 14-item questionnaire developed by Gao et al. [21]. It was customized for our research based on Iranian experts’ views. The questionnaire was reviewed in several focus group discussions. As a result, unnecessary items that were not applied to Iranian twins were removed based on the opinions of experts. Consequently, mothers were asked to respond to each question according to the provided options.

All of the questions were translated into Persians. The internal content validity of our questionnaire was confirmed by those experts who were familiar with our concept and participated in all focus group discussions. Reliability is related to the ability of an instrument to measure consistently. The internal consistency was assessed using Cronbach's alpha [22].

To validate the results of the zygosity questionnaire, we have a plan for the second phase. All parents that give us informed consent were invited for their child’s health evaluation in the twins’ health promotion clinic (#Project No: 98–3-252–46,014). In this project at the time of the twin's visit, one of the researcher’s team members will ask all questions from the mothers. Also, blood samples will be sent to the Genetic lab for zygosity determination in volunteered twins. The validity of the in-home filled questionnaire will be determined by comparison to the researcher-filled questionnaire in all twins visited at the health promotion clinic and the genetic results of volunteered twins.

### Analysis

Statistical analysis was performed using SPSSv.20. Through descriptive analysis, frequencies and percentages were calculated for categorical variables and means and standard deviations for continuous variables. Bivariate correlation analysis was first used to check the zero-order correlations between all the variables. To analyze the similarity questionnaire results based on the Pea-in-pod zygosity score, logistic regression could be selected to model zygosity based on the variables in the similarity questionnaire because the target value (zygosity) is a binary variable. The correlation between these two questionnaires and finding out the screening criteria for zygosity is the subject of further study.

## Results

### Expert panel

The expert panel comprises 16 experts comprises whose demographic characteristics are listed in Table 1. The mean age of all participants was almost 43 ± 1.54, while sixty percent of participants were female. All of the invited experts attended all focus group meetings. All proposed data elements and viewpoints were transcripted by two researchers. The data saturation occurred when no new data elements were suggested, and the data elements were formed.

**Table 1:** Demographic characteristics of the expert panel

	**Data**	**Frequency**	**Percentage**
**Specialist**	Internist	4	25.00%
	Pediatricians	4	25.00%
	Psychiatrist	3	18.75%
	Community Medicine	1	6.25%
	Epidemiologists	2	12.50%
	Oral disease specialist	1	6.25%
	Medical informatician	1	6.25%
**Age**	30–45	6	37.50%
	45–60	5	31.25%
	> 60	4	25.00%
**Experience**	< 5yrs	5	31.25%
	5–10 yrs	3	18.75%
	> 10 yrs	6	37.50%

### Database design and data collection method development

Based on summarizing the results of focus group discussions, two levels and eight sub-levels of data elements were determined to store the final data sets in our school-aged twin registry study. All of the potential datasets were categorized into two main themes, the first level of data including essential datasets, and the second level of data including medical history, general health, and clinical data. Data elements approved by more than 85% of experts were identified as the final data elements. In total, 52 data items were recognized as essential data sets. The details of the results of this step will be explained in another article. These two levels of data are described in the following in Table 2. The data collection protocol was also formed as a result of group discussions, which was explained in the form of the twin registration protocol in the method section.

**Table 2:** Details of data elements items

***The first level of data (23 data items)***	**Demographic data of twin pairs (14 data items):** First name and surname, Age, Birthdate, Type of twins (twin, triplet, quadruplet), Sex, Gender, City, Contact info, Date of data collection, Region of school, Education level, Height, Weight, BMI **Demographic data of parents (Seven data items):** Mother’s Name, Father’s name, education level of parents, Occupation, Mother’s age in delivery time, mode of delivery, Smoking habits **Zygosity info (Two items):** Zygosity information based on questionaries, Physical similarity questionaries score
*The second level of data (29 data items)*	**Illness history in the family (One data item)**: History of chronic illness or disorder in the family **History of any disorder or illness in infancy (Six data elements)**: History of hospitalization in infancy, history of hospitalization in NICU, History of surgery in infancy, History of pulmonary disorders in infancy, History of nervous system disorder in infancy, Drug usage in infancy **History of specific or chronic diseases in the post-neonatal period (11 data elements):** Appearance disorders, CNS disorders, Head & neck problems, pulmonary disease or disorder, Cardiology problems, Hematology disease, Urogenital disease, Gastrointestinal, Endocrine disease or disorder, Musculoskeletal disorders, Any type of skin disease, Other chronic diseases **Mental health status (Two data elements):** History of behavioral or mental disorders, General Health Questionnaire (GHQ) result **General Health Status of twins (Eight data elements):** Present medical history**,** Symptoms of present illness**,** History of hospitalization, History of surgery, Drugs, Special habits or addiction, Clinical examinations, Assessment **DNA or biological samples**

The experts concluded that the first level of data should be collected in the preliminary phase with the cooperation of schools, and the second level of data, which is related to the general health of twins, should be collected in the second phase with the in-person visit of the experts. The plan for setting up health promotion clinics to improve the health of twins with the cooperation of clinicians affiliated with the Tehran University of Medical Sciences was also approved at this stage.

### Estimation of the population of twin students across the country

**Fig. 2:**
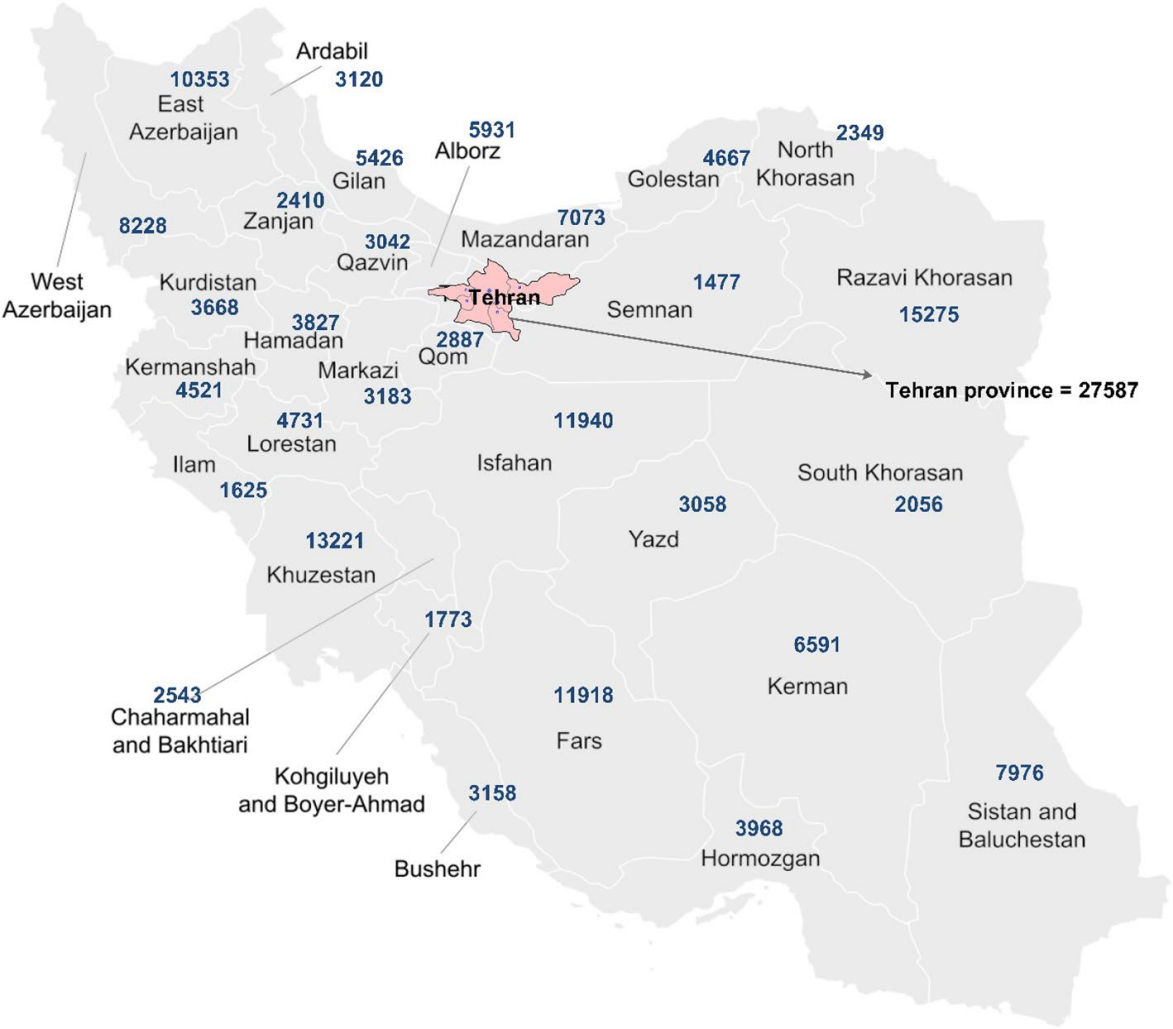
The distribution of school-aged individual twins in Iran

We describe the frequency of twin populations throughout Iran in this section based on retrieved information from the Ministry of Education database. In total, the initial information of 189,738 students was retrieved from the database. All of them were twins who lived in 31 provinces of Iran. The number of school-aged twins based on each province is depicted on the map of Iran in Fig. 2.

**Fig. 3:**
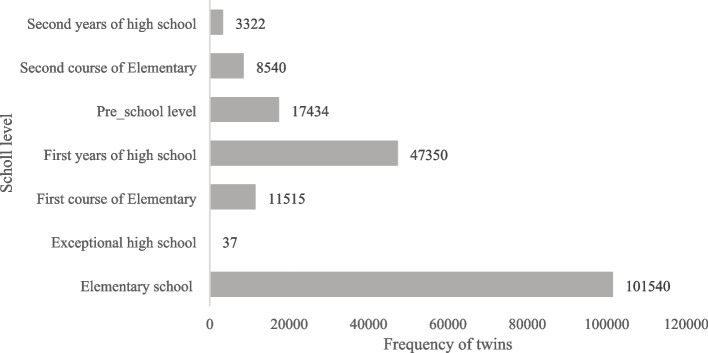
The distribution of twins based on the education level

The age range of all recovered twins is between seven and 19 years with a mean age of 13.46 (± 2.942) while 16% of the twins were 13 years old. All the twins were born between 2003 and 2017, of which 94,997 are boys (50.1%) and 94,741 are girls (49.9%). Of these, 90,522 were twins, 2,603 were triplets, and 353 were quadruplets or higher. Of 189,738 twin students, 2734 reported that they suffer from physical disabilities (1.4%). The distribution of school-aged twins is described in Figs. 2 and 3 in Iran. The majority of twins were at the elementary level (73.2%).

### Preliminary results based on gathered data

**Fig. 4:**
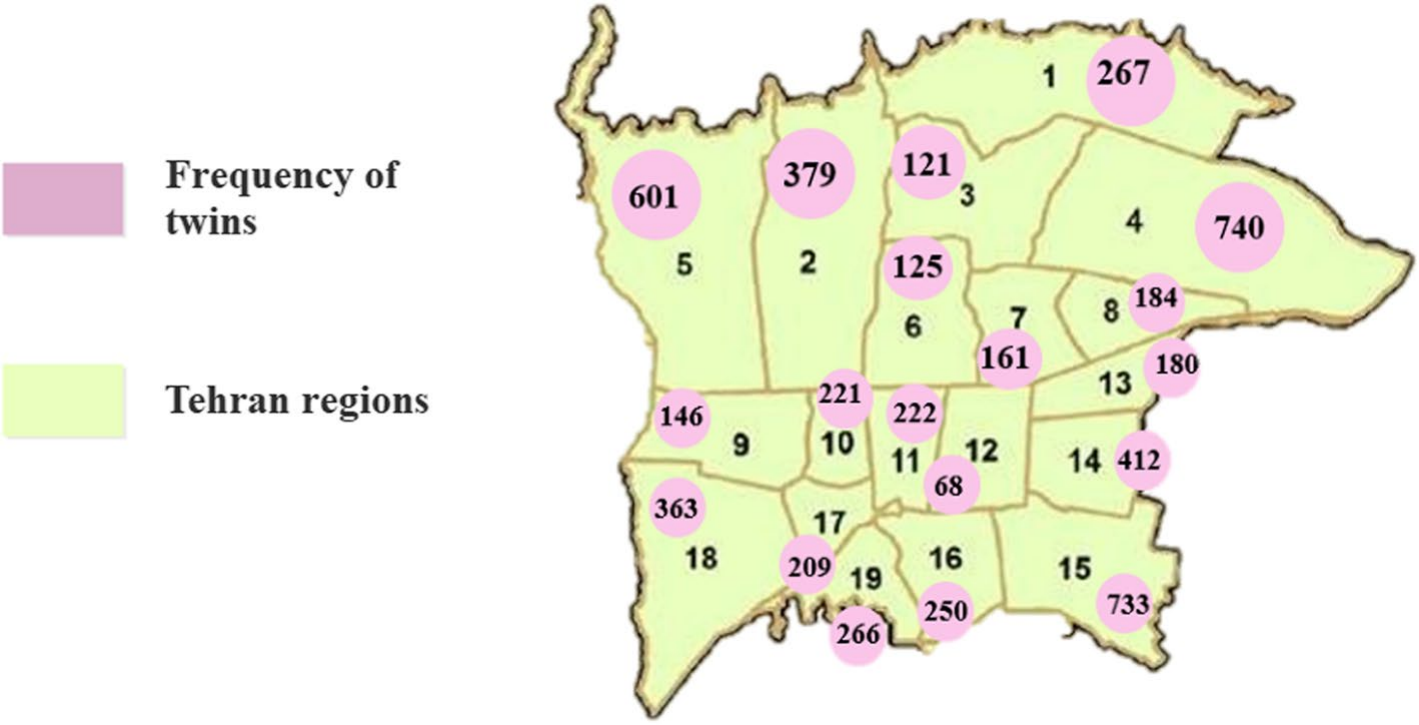
The distribution of twin pairs in nineteen regions of Tehran

The first phase of our program started in 2018 and data collection continued until 2022. Of 27,587 school-aged twins living in Tehran province, 17,208 individual twins live in Tehran city according to retrieved data from the Ministry of education. Of them, a total of 5,642 pairs of school-aged twins agreed to participate in this program and gave us informed consent to contact them. The response rate was almost 72%. All of them provide us with their demographic data. The frequency of twin pairs in different regions of Tehran is shown on the map Tehran in Fig. 4. The mean age of mothers was 44.231 ± 6.287.

**Fig. 5:**
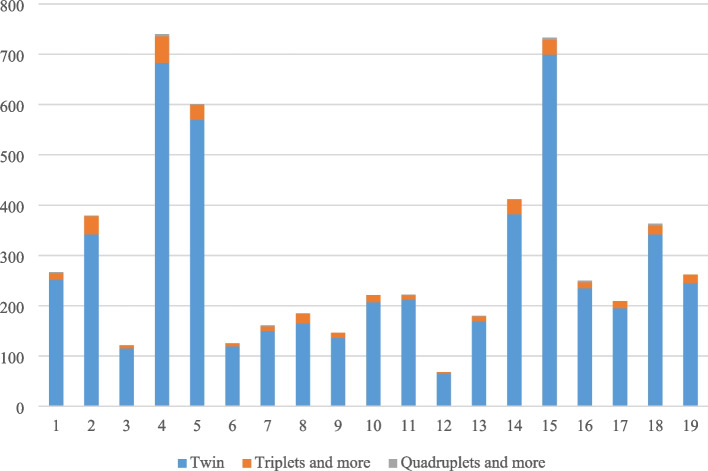
The frequency of Twin types in Tehran city

According to the recorded information and the results of questionnaires, our sample size comprised 9772 twins, 906 triples, and 92 quadruplets. The frequency of twins according to the type of multiple twins in different areas of Tehran is also shown in Fig. 5.

Hence, the information of 10,770 individual twins has been enrolled in our registry. The mean age of twins is 14.38 ± 3.337. Of them, the majority of school-aged twins (63%) studied in elementary schools. Of 5,642 twin pairs who signed the informed consent, all of them fill-out the first part of the questionnaire regarding their demographic information but only 4988 (88.84% of all contacted twin pairs) twin mothers fill-out zygosity and physical similarity questionnaires completely, and accurately. The results showed that all of them were born between 2002 and 2013. The results showed that 42.6 percent of parents of recorded twins have academic educations, while only two parents were illiterate.

### Findings from the questionnaires in the first phase

The content reliability of the Persian version of the Pea-in-Pods questionnaire has been confirmed by experts who participated in focus group discussions through this survey. The analysis of the filled questionnaire showed that it had adequate internal consistency in this study (Cronbach’s α = 0.923). Accordingly, the physical similarity questionnaire (11 items) had a Cronbach’s alpha of 0.936 and it reached a high value.

To calculate the zygosity score, only those who answered all three questions of the questionnaire remained. We determined the zygosity based on the overall score defined by Ooki. If the overall score is less than 6 or greater than 3, the twin was considered monozygotic (MZ). Accordingly, if the overall score is less than 10 or greater than 6, the twin was considered dizygotic (DZ) [23]. The results of zygosity determination according to the above formula and the first three questions are described in Table 3. Approximately one percent of the twins did not specify their twin types.

**Table 3:** Frequency of monozygotic and dizygotic based on Pea-in-Pods questionnaire

		***Twin Type***				
		**Quadruplet**	**Twins**	**Triplet**	**All participants**	**Total**
***MZ***	**Not Defined**	0	5	0	5	**703 (14%)**
	**M-M**	0	247	1	248	
	**F-F**	0	440	1	441	
	**More than two pairs**	0	0	9	9	
***DZ***	**Not Defined**	0	21	1	22	**4285 (86%)**
	**M-M**	0	1108	5	1113	
	**F-M**	0	1155	9	1164	
	**F-F**	0	1698	5	1703	
	**More**	22	0	261	283	

*M-M* Male-Male, *F-F* Female-Female, *M-F* Male, Female

The second part of our questionnaire was determined to 11 items concerning physical similarity which was understood by mothers. All of the questions asked ranged from “no difference” to “completely different”. This part of the questionnaire is scored based on the difference with a score of one to three. According to the answers, we analyzed the 11 items concerning physical similarity based on cross-tabulation. According to the score devoted to each question in the physical similarity questionnaire, the overall score for each characteristic of twins is calculated. The results showed that all of the scores ranged from 11 to 33. In this questionnaire, the total scores are lower in identical twins than in non-identical twins.

The results of different degrees of similarity according to results in monozygotic and dizygotic twin pairs are described in Table 4. These responses were expressed by mothers. This part of the questionnaire is filled out based on the cognitive understanding that mothers have about their children.

**Table 4:** The percentage of physical similarity in MZ and DZ twins based on the mother’s perception

		**No response**	**No difference**	**Somewhat similar**	**Completely different**
**Facial appearance**	**MZ**	10	293	387	13
	**DZ**	21	161	1508	2595
**Position of the hair whorl**	**MZ**	2	464	211	26
	**DZ**	20	677	1803	1785
**Shape of eyebrow**	**MZ**	4	489	188	22
	**DZ**	26	519	1371	2369
**Shape of eyes**	**MZ**	5	393	279	26
	**DZ**	26	379	1353	2527
**Shape of ears**	**MZ**	5	536	141	21
	**DZ**	43	717	1581	1944
**Voice**	**MZ**	7	212	401	83
	**DZ**	23	311	1422	2529
**The number of moles or spots**	**MZ**	13	129	339	222
	**DZ**	87	186	898	3114
**Shape of fingers**	**MZ**	4	496	176	27
	**DZ**	35	476	1093	2681
**Sleeping face**	**MZ**	6	487	182	28
	**DZ**	40	493	1600	2152
**Sleeping posture**	**MZ**	2	438	222	41
	**DZ**	35	575	1592	2083
**The tendency of concordance for illness**	**MZ**	7	342	290	64
	**DZ**	40	647	2018	1580

### Analysis

The comparison of the outcomes showed that there is a correlation between the total score obtained from the physical similarities with the zygosity score from the pea-in-pods questionnaire. The zygosity score and total score of similarity have a normal distribution. The zygosity score had a mild correlation with the total score of similarity (*r* = 0.579) using the pearson correlations coefficient. If we conduct a Spearman Correlation Coefficient on twins of the same sex, the zygosity score had a strong correlation with the total score of similarity (*r* = 0.696). All eleven variables of the similarity questionnaire showed a positive correlation with zygosity scores, and the associations with zygosity were statistically significant. Zero-order correlations between the variables are shown in Table A-2, Appendix A-1.

A logistic regression modeling was performed to model the zygosity based on the results of the similarity questionnaire. Logistic regression was successfully developed with 87.5% accuracy, 95.8% sensitivity, and 33.5% specificity. The results are shown in Table 5.

**Table 5:** Result of logistic regression

Factors	B	S.E	Wald	Sig	OR	95% C.I
Facial appearance	.542	.095	32.401	< 0.001	1.719	1.427 to 2.072
Position of the hair whorl	.048	.090	.287	0.592	1.049	.880 to 1.250
Shape of eyebrow	.403	.095	17.797	< 0.001	1.496	1.241 to 1.804
Shape of eyes	.307	.089	12.007	< 0.05	1.360	1.143 to 1.618
Shape of ears	.284	.091	9.739	< 0.05	1.329	1.111 to 1.588
Voice	-.137	.081	2.863	< 0.05	.872	.744 to 1.022
The number of moles or spots	-.335	.073	20.971	< 0.001	.715	.620 to 0.826
Shape of fingers	.596	.086	47.450	< 0.001	1.815	1.532 to 2.150
Sleeping face	.477	.098	23.718	< 0.001	1.612	1.330 to 1.953
Sleeping posture	.134	.090	2.217	0.137	1.143	.959 to 1.363
The tendency of concordance for illness	-.210	.077	7.319	< 0.001	.811	.697 to 0.944

**Fig. 6:**
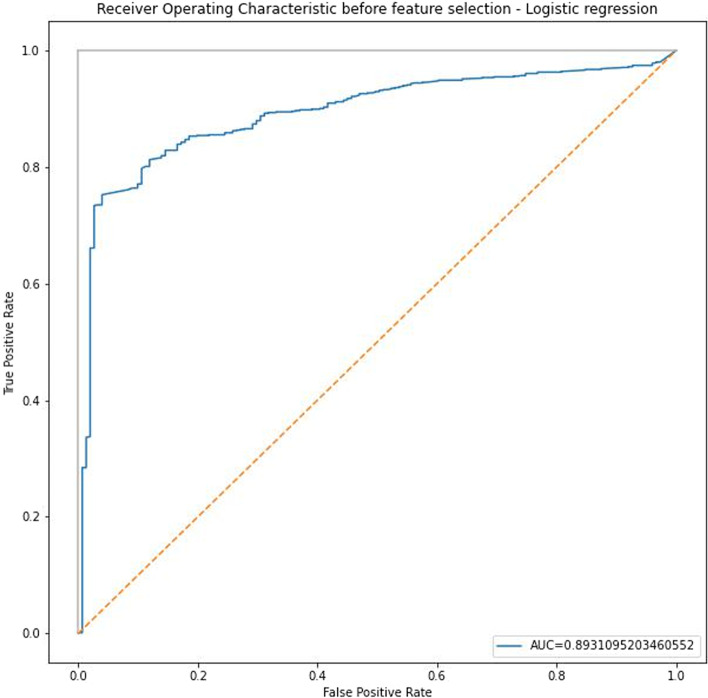
Receiver operating characteristic curve for logistic regression analysis

The results showed that all of the variables have a significant correlation with zygosity except sleeping posture and hair whorl. Since the accuracy doesn’t show the performance of the model alone, the ROC curve (receiver operating characteristic curve) was calculated for the developed model. The ROC curve for logistic regression was 89.31 (Fig. 6).

## Discussion

In this project, the initial steps and progress of the Iranian school-aged twin registry are described. Our school-aged twin registry will be covered all of the monozygotic (MZ) and dizygotic (DZ) twin students with different educational degrees in collaboration with the Ministry of Education. Tehran as the most urban city in Iran with a population of 8,693,706 people was selected for the first phase of the study. Education in Iran is mandatory at primary school age. On other levels, students must be registered in the Iranian education system even if they study at home to obtain the educational certificate. Hence, we succeeded to get the complete list of student twins with the cooperation of the Ministry of Education. As a result, in collaboration with the Iranian ministry of health and the well-designed educational system, we succeeded to record more than 5,000 pairs of twins. This number seems significant compared to similar studies conducted in other countries [24, 25].

Evidence showed that twin registries failed without a continuous health follow-up program [9]. Therefore, in the first step, we defined the main aspects of our program and enrollment process to address this challenge. With predefined datasets, all of the information was recorded in a structured way to avoid problems with data redundancy and improper information recording [26]. Defining a uniform set of data could enhance data collection quality and it is so effective in data retrieval and analysis [27].

Due to the absence of genotyping information in the initial phase, we utilized the PPQ questionnaire as an indicator of zygosity in our study. Jarrar also believes that in the absence of access to biological samples, this questionnaire is the best indicator to determine the degree of homogeneity in twins [28]. Besides, in cooperation with the parents of the twins, we succeed to collect the results of zygosity recognition questionnaires in addition to recording demographic information. However, we will compare the results of this study to the results of analyzing the biological samples for zygosity determination.

The effectiveness of zygosity diagnosis by this questionnaire has been proven in twin registry-based research [18, 20, 21, 29, 30]. The conducted studies showed that such a questionnaire can be administered over the telephone and can be filled out by mothers of twins. According to the formula represented by Ooki [31], the zygosity of more than ninety percent of twins was correctly diagnosed. After that, this cutting point was employed by famous twin registries for zygosity determination such as the Australian twin registry [32], Serbian twin studies [33], TwinsUK registry [28], Chinese Adolescent Twin study [21, 34], Japanese twin registry [18], and Washington State twin registry [23].

The number of identical twins by the zygosity questionnaire is low compared to most previous twin studies. The results of our study are consistent with a previous multicenter study on multiple births conducted on the frequency of multiple twins in Tehran in which the rate of dizygotic twins in multiple births was high [35]. In addition to Iran, the high rate of DZ twinning has also been seen in Western Nigeria which was high compared to the European population [36]. Our study also showed that the average reproductive age of mothers of school-aged twins is almost 35.5 years. It has been supposed that increasing the reproductive age of women, ovulation with fertility drugs in women over 30 years old, and the emergence of ART techniques can be effective in increasing the birth of non-identical multiples [37]. Also, Zygosity questionnaires results should be verified in the next phase of the study by biobank samples,

Reliable and sustained participation was an imperative limitation in registry-based studies [38]. Getting involved participants in such studies is complicated in some circumstances. The analysis of similar twin registries revealed that developing registries only for epidemiological intention has not been successful [9]. The development of registry-based studies should contain a comprehensive follow-up program like longitudinal studies. Thus, developing a longitudinal twin health promotion program to assess the physical and mental health of school-aged twins is the future goal of our registry. Devising the school-aged twins' physical and mental health follow-up programs could be considered the new solution to address the potential challenges. Establishing a health promotion clinic in collaboration with clinicians interested in twin studies is one of the plans of this project. Establishing such a clinic for long-term monitoring of twins' health can provide the possibility of collecting information related to physical and mental health easily. Such programs can also make them interested in participating in this program due to individual benefits for twins.

Another limitation of our project was voluntary participation. Thus, the number of participants was limited to those who were willing to provide us with their children's information. The next limitation that we can mention was related to pea-in-pods questionnaire. To calculate the zygosity, all three questions of the pea-in-pods questionnaire had to be answered to determine zygosity. As a result, questionnaires that did not answer all three questions were excluded from the final analysis. Moreover, the validity of our results and our questionnaires will be determined in the next phase.

## Conclusion

This paper described the initial achievements, methods, and initial results of a school-based twin registry. The experiences of our twin registry researchers showed that developing a twin registry with only population-based objectives is not successful. Recruiting school-aged twins through school health assistants leads to high enrollment and decreasing costs for the twin registry. The study showed a high rate of dizygotic twins that need to be verified by twin bio-sample in the next phase of studies.

Future articles are planned to describe the comprehensive conceptual model for developing health promotion clinics to assess the general physical and mental health of twins. Such studies not only enhance the general health of children but also leads to the creation of a large database of twin data for further research.

## Supplementary Information

**Additional file 1.**

**Additional file 2.**

**Additional file 3.**

## Data Availability

The datasets generated and/or analyzed during the current study are not publicly available but are available from the corresponding author at reasonable request.
