# Supplementary material for: Iranian school-aged twin registry: preliminary reports and project progress
Source: BMC Pediatr. 2023 Feb 10;23:71. doi: 10.1186/s12887-023-03865-x (PMC9912495; doi:10.1186/s12887-023-03865-x)
Supplement: Supplementary file 1 — Additional file 1. [file 12887_2023_3865_MOESM1_ESM.docx]

**Appendix A-1:**

Table A-1- Sum-up score of physical similarity questionnaire based on zygosity determined by the first three questions

|  | **MZ** | **DZ** |
| --- | --- | --- |
| 11 | 33 | 13 |
| 12 | 55 | 19 |
| 13 | 78 | 50 |
| 14 | 77 | 43 |
| 15 | 105 | 50 |
| 16 | 79 | 85 |
| 17 | 47 | 63 |
| 18 | 42 | 86 |
| 19 | 39 | 82 |
| 20 | 25 | 90 |
| 21 | 20 | 93 |
| 22 | 32 | 171 |
| 23 | 25 | 210 |
| 24 | 22 | 210 |
| 25 | 11 | 228 |
| 26 | 2 | 284 |
| 27 | 3 | 317 |
| 28 | 1 | 400 |
| 29 | 1 | 368 |
| 30 | 3 | 379 |
| 31 | 0 | 312 |
| 32 | 1 | 312 |
| 33 | 2 | 420 |
|  | 703 | 4285 |

Table A-2. Pearson’s correlation coefficients between all variables in the similarity questionnaire and zygosity score

|  | | **Zygosity** | **Face** | **Hair** | **Eyebrow** | **Eyes** | **Ears** | **Voice** | **Moles** | **Finger** | **Sleep Face** | **Sleep Body** | **Illness** |
| --- | --- | --- | --- | --- | --- | --- | --- | --- | --- | --- | --- | --- | --- |
| **Zygosity** | **Correlation** | 1 | .504 | .396 | .478 | .471 | .431 | .354 | .271 | .505 | .459 | .402 | .288 |
|  | **Sig. (2-tailed)** |  | <0.01 | <0.01 | <0.01 | <0.01 | <0.01 | <0.01 | <0.01 | <0.01 | <0.01 | <0.01 | <0.01 |
| **Face** | **Correlation** | .504 | 1 | .539 | .630 | .616 | .560 | .461 | .340 | .607 | .583 | .494 | .367 |
|  | **Sig. (2-tailed)** | <0.01 |  | <0.01 | <0.01 | <0.01 | <0.01 | <0.01 | <0.01 | <0.01 | <0.01 | <0.01 | <0.01 |
| **Hair** | **Correlation** | .396 | .539 | 1 | .601 | .514 | .530 | .384 | .300 | .526 | .503 | .457 | .361 |
|  | **Sig. (2-tailed)** | <0.01 | <0.01 |  | <0.01 | <0.01 | <0.01 | <0.01 | <0.01 | <0.01 | <0.01 | <0.01 | <0.01 |
| **Eyebrow** | **Correlation** | .478 | .630 | .601 | 1 | .655 | .608 | .439 | .331 | .635 | .571 | .509 | .381 |
|  | **Sig. (2-tailed)** | <0.01 | <0.01 | <0.01 |  | <0.01 | <0.01 | <0.01 | <0.01 | <0.01 | <0.01 | <0.01 | <0.01 |
| **Eyes** | **Correlation** | .471 | .616 | .514 | .655 | 1 | .592 | .442 | .328 | .587 | .534 | .467 | .350 |
|  | **Sig. (2-tailed)** | <0.01 | <0.01 | <0.01 | <0.01 |  | <0.01 | <0.01 | <0.01 | <0.01 | <0.01 | <0.01 | <0.01 |
| **Ears** | **Correlation** | .431 | .560 | .530 | .608 | .592 | 1 | .446 | .322 | .594 | .550 | .485 | .373 |
|  | **Sig. (2-tailed)** | <0.01 | <0.01 | <0.01 | <0.01 | .000 |  | <0.01 | <0.01 | <0.01 | <0.01 | <0.01 | <0.01 |
| **Voice** | **Correlation** | .354 | .461 | .384 | .439 | .442 | .446 | 1 | .315 | .467 | .451 | .416 | .308 |
|  | **Sig. (2-tailed)** | <0.01 | <0.01 | <0.01 | <0.01 | .000 | .000 |  | <0.01 | <0.01 | <0.01 | <0.01 | <0.01 |
| **Moles** | **Correlation** | .271 | .340 | .300 | .331 | .328 | .322 | .315 | 1 | .379 | .374 | .350 | .289 |
|  | **Sig. (2-tailed)** | <0.01 | <0.01 | <0.01 | <0.01 | <0.01 | <0.01 | <0.01 |  | <0.01 | <0.01 | <0.01 | <0.01 |
| **Finger** | **Correlation** | .505 | .607 | .526 | .635 | .587 | .594 | .467 | .379 | 1 | .623 | .544 | .389 |
|  | **Sig. (2-tailed)** | <0.01 | <0.01 | <0.01 | <0.01 | <0.01 | <0.01 | <0.01 | <0.01 |  | <0.01 | <0.01 | <0.01 |
| **Sleep Face** | **Correlation** | .459 | .583 | .503 | .571 | .534 | .550 | .451 | .374 | .623 | 1 | .677 | .425 |
|  | **Sig. (2-tailed)** | <0.01 | <0.01 | <0.01 | <0.01 | <0.01 | <0.01 | <0.01 | <0.01 | <0.01 |  | <0.01 | <0.01 |
| **Sleep Body** | **Correlation** | .402 | .494 | .457 | .509 | .467 | .485 | .416 | .350 | .544 | .677 | 1 | .450 |
|  | **Sig. (2-tailed)** | <0.01 | <0.01 | <0.01 | <0.01 | <0.01 | <0.01 | <0.01 | <0.01 | <0.01 | <0.01 |  | <0.01 |
| **Illness** | **Correlation** | .288 | .367 | .361 | .381 | .350 | .373 | .308 | .289 | .389 | .425 | .450 | 1 |
|  | **Sig. (2-tailed)** | <0.01 | <0.01 | <0.01 | <0.01 | <0.01 | <0.01 | <0.01 | <0.01 | <0.01 | <0.01 | <0.01 |  |
| * Correlation is significant at the 0.01 level (2-tailed). | | | | | | | | | | | | | |
