# Supplementary material for: Iranian school-aged twin registry: preliminary reports and project progress
Source: BMC Pediatr. 2023 Feb 10;23:71. doi: 10.1186/s12887-023-03865-x (PMC9912495; doi:10.1186/s12887-023-03865-x)
Supplement: Supplementary file 2 — Additional file 2. [file 12887_2023_3865_MOESM2_ESM.docx]

**Appendix A-2- Feature importance using data mining techniques**

Applying data mining algorithms can find the most important features based on the data set [1]. To analyze the similarity questionnaire results based on the Pea-in-pod zygosity score, a data mining algorithm was applied to reveal the most effective variables in twins’ similarity.

We applied a supervised XGBoost model due to its ability to feature selection in a huge amount of data [2, 3]. In the first phase, pre-processing and cleaning data were conducted. Pre-processing includes removing missed values and converting string variables to ordinal values. In the second phase, all of the data was split into trained and tested data. XGBoost trained with trained data and the final predictor was created based on test data. Finally, the most important features were ranked by Multi-tree XGBoost according to their importance [4]. Thus, Shapley Additive Explanations (SHAP) in combination with Multi-tree XGBoost was employed to determine the most important variables in the second questionnaire [5, 6]. Therefore, a ranking selection consisting of iteratively dropping the less important features was conducted while retraining the model until convergence is reached. Finally, the most important similarity variables in identical twins were extracted in order of importance based on the results of the questionnaires.

As described in the method section, we utilized data mining techniques such as the SHAP method based on the XGboost method to find more important features regarding the degree of similarity and zygosity. SHAP features are important because they provide an alternative to describing an object, using its most important characteristics, and reducing the amount of information stored. The ranking of variables is shown in Fig A-2-1 on the left side. On the right side of Fig, A-2-1, a density scatters plot of SHAP values that integrates feature importance is explained too. A SHAP analysis presents the employed features visually with an indication of the influence on the outcome: within each line, higher values are represented in red and lower in blue. All of the variables are shown in the plot according to their importance on the left side. According to SHAP methods, all of the variables that have a positive score (greater than zero) could be effective in the final prediction model. The related variables are sorted in Fig A-2-1 based on their importance.

The results showed that the shape of fingers, facial appearance, the shape of the eyebrow, and sleeping face include the most important features which are most similar in identical twins.

| 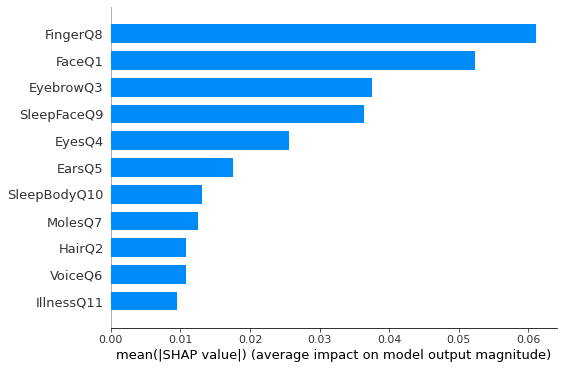 | 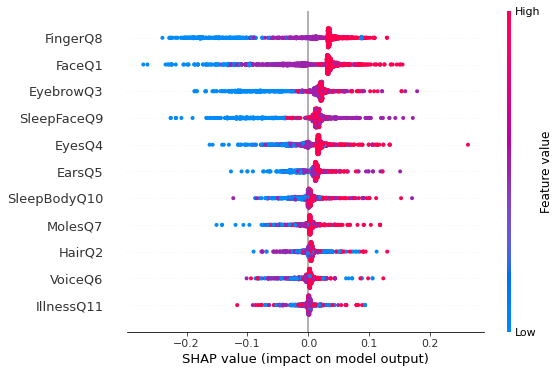 |
| --- | --- |

Fig A-2-1. (Left) SHAP summary plots for the 11 most important features under their mean absolute values. Beeswarm plot (Right), where each dot corresponds to each twin, showing the effect of the feature on the model’s prediction for zygosity.

After feature selection, the logistic regression was conducted using the top 10 variables selected by the XGBoost method again. Logistic regression was developed based on the most important features with 90 % accuracy. Since the accuracy doesn’t show the performance of the model alone, the ROC curve (receiver operating characteristic curve) was calculated for the developed model. The ROC curve for logistic regression after feature engineering reached 91.92. A comparison of two developed models before and after feature selection based on the ROC diagram is shown in Fig A-2-2. The results showed that the overall performance of the developed model could be improved using feature engineering approaches.

| 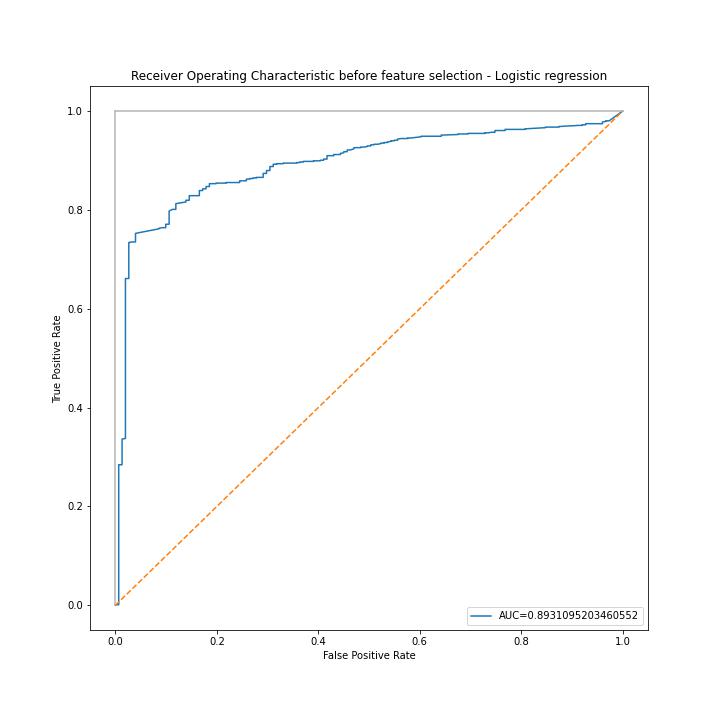 | 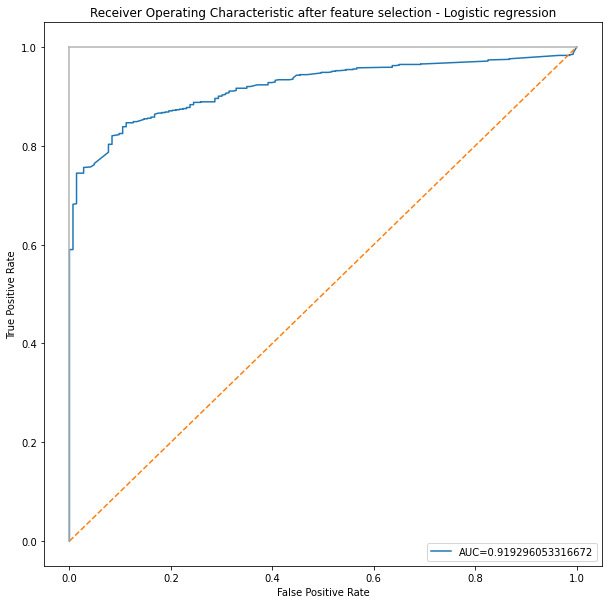 |
| --- | --- |

Fig A-2-2. Comparing the results of the logistic regression analysis before and after feature selection

1. Safdari R, Rezayi S, Saeedi S, Tanhapour M, Gholamzadeh M: **Using data mining techniques to fight and control epidemics: A scoping review**. *Health and Technology* 2021, **11**(4):759-771.

2. Ogunleye A, Wang Q-G: **XGBoost model for chronic kidney disease diagnosis**. *IEEE/ACM transactions on computational biology and bioinformatics* 2019, **17**(6):2131-2140.

3. Alsahaf A, Petkov N, Shenoy V, Azzopardi G: **A framework for feature selection through boosting**. *Expert Systems with Applications* 2022, **187**:115895.

4. Chowdhury ME, Rahman T, Khandakar A, Al-Madeed S, Zughaier SM, Doi SA, Hassen H, Islam MT: **An early warning tool for predicting mortality risk of COVID-19 patients using machine learning**. *Cognitive Computation* 2021:1-16.

5. AlJame M, Ahmad I, Imtiaz A, Mohammed A: **Ensemble learning model for diagnosing COVID-19 from routine blood tests**. *Informatics in Medicine Unlocked* 2020, **21**:100449.

6. Domínguez-Olmedo JL, Gragera-Martínez Á, Mata J, Pachón Álvarez V: **Machine Learning Applied to Clinical Laboratory Data in Spain for COVID-19 Outcome Prediction: Model Development and Validation**. *J Med Internet Res* 2021, **23**(4):e26211.
